# Adapting the modified WHO safety checklist for non-operating room anesthesia: A Quality improvement project

**DOI:** 10.12669/pjms.42.1.13160

**Published:** 2026-01

**Authors:** Faraz Mansoor

**Affiliations:** Dr. Faraz Mansoor, FCPS (Anesthesia), FCPS (Critical Care Medicine) Consultant Anesthetist, Department of Anesthesia, Shaukat Khanum Memorial Cancer Hospital and Research Center, Peshawar, Pakistan

**Keywords:** Clinical Audit, WHO Patient Safety, Radiology, Safety checklist, Sedation

## Abstract

**Background & Objective:**

Patient safety checklists are established tools for reducing preventable errors in clinical practice. The use of World Health Organization (WHO) safety checklist outside the operating room environment remains inconsistent. This audit was conducted to assess compliance with the Royal College of Radiologists (RCR) standards.

**Methodology:**

A prospective audit by the anesthesia team was carried out in the Radiology Department of Shaukat Khanum Memorial Cancer Hospital and Research Center, Peshawar. All patients receiving sedation for CT, MRI, or PET scans in February 2023 were included (n=30). Data sources included patient records, resuscitation equipment logs, drug inventory checks, staff training records, and direct observations. Standards were benchmarked against Royal College of Radiologists (RCR) recommendations. Audit findings were presented to the Quality Council, and a modified WHO safety checklist specific to radiology was developed and implemented. A re-audit was latter conducted in March–April 2024 which included 55 patients.

**Results:**

Baseline data demonstrated compliance with all RCR standards except for the use of a safety checklist, which was absent in all 30 cases. In response, a concise modified WHO checklist was introduced, incorporating high-risk patient identification and independent double-checking of medications. The re-audit showed 100% compliance across all RCR indicators, including checklist use, in 55 patients.

**Conclusion:**

The adaptation of the modified WHO safety checklist for radiology enhanced compliance with international safety standards and closed previously identified gaps. Regular clinical audits and the incorporation of tailored checklists can strengthen patient safety in non-operating room anesthesia.

## INTRODUCTION

In recent years, there has been a notable increase in both the volume and complexity of patients undergoing medical procedures outside the confines of the operating theater. Establishing a culture of safety is crucial for mitigating complications associated with the administration of anesthesia and sedation in these settings. The Francis Report emphasized the importance of standards, audit, and training, stating that “no provider should deliver any service that does not comply with fundamental standards of care.” It further stressed that these standards must be evidence-based, measurable, and designed to ensure safe and effective service delivery.^1^

Medical errors are preventable through the development of robust systems and the promotion of an improved organizational safety culture.^2^ Unfortunately, in Pakistan, patient safety data remain limited, and the frequency with which healthcare institutions engage in departmental quality improvement activities, particularly in high-risk services, is uncertain. Moreover, no comprehensive national database exists to track unanticipated morbidity and mortality. The use of structured patient safety checklists outside the operating theater is not yet routine practice, although available evidence suggests that their implementation can improve patient outcomes.^3^

Following the inauguration of new operation theaters at Shaukat Khanum Hospital Peshawar center, anesthesia services were extended to radiology and endoscopy suites. In line with institutional commitment to quality improvement, annual audits were initiated in accordance with recommendations from the Royal College of Anaesthetists and the Royal College of Radiologists.^4^ The present audit was conducted to identify deficiencies in current practice and to guide interventions for strengthening patient safety during sedation and anesthesia outside the operating theater.

### Safety checklists in anesthesia:

A checklist can be defined as an aid that is employed in the execution of repetitive tasks, decreasing the risk of failure by addressing potential limitations associated with human memory and attention. These checklists are particularly useful for the tasks involving safety to ensure that everything is done correctly.^5^ Historically, the first most widely accepted checklist in medicine was introduced for the central venous catheter insertion.^6^ In order to ascertain the fulfillment of the safety checklist objectives, it is important that the elements subject to scrutiny are evidence-based. Based upon robust scientific evidence, a considerable number of safety checklists have found their application in the field of anesthesia.

More importantly, the Royal College of Anaesthetists, in its Guidelines for the Provision of Anaesthesia Services in the Non-theatre Environment, mandates compliance with the WHO Surgical Safety Checklist in all areas where anesthesia is administered outside the operating theatre, including radiology and endoscopy suites.^7^ Historically, the implementation of a checklist for central venous catheterization has demonstrated a 66 % reduction in the incidence of catheter-related bloodstream infections. Recently, Bielka and colleagues demonstrated a significant improvement in the outcome after the application of WHO Surgical Safety Checklist and the Anesthesia Equipment Checklist.^8^ There is evidence that the utilization of safety checklists holds the capacity to identify potential issues in a considerable proportion of cases, thereby contributing to informed decision-making and provision of safe patient care.^9^,^10^ At present there is a long list of patient safety checklists that are being used in the clinical practice.

These anesthesia patient safety checklists play a key role in ensuring thorough preoperative, intraoperative, and postoperative care. Some of the integral components encompass patient identification, meticulous airway assessment, rigorous equipment verification, safe medication management, and diligent monitoring. These checklists serve as systematic tools, mitigating errors, fostering effective communication, and elevating overall patient safety standards during anesthesia administration and throughout the perioperative period.^10^ Their role in anesthesia practice is well established.

### Baseline data:

Regular clinical audits are an integral part of quality improvement and patient safety within healthcare systems. Both the Royal College of Radiologists (RCR) and the Royal College of Anaesthetists emphasize the importance of conducting annual audits in Radiology to ensure the safe delivery of care in geographically remote and high-risk areas of the hospital. At Shaukat Khanum Memorial Cancer Hospital and Research Center, Peshawar, anesthesia services were inaugurated in April 2021. In February 2023, the department undertook its first audit in the Radiology unit, focusing on patients undergoing sedation. It is noteworthy that, at the time of this audit, an MRI-compatible anesthesia machine was not available.

This prospective audit was conducted in accordance with RCR recommendations.^4^ All patients scheduled for CT, MRI, and PET scans on the selected audit days were included. The audit assessed patient records, resuscitation equipment logs, and drug inventories. Additionally, training records of the relevant staff were reviewed, and real-time observations were made during the provision of sedation. Approval for this clinical audit was obtained from the institutional clinical audit committee.

A total of 30 patients (n = 30) received sedation for different radiological procedures during the audit period: 15 for computed tomography (CT), 5 for positron emission tomography (PET), and 10 for magnetic resonance imaging (MRI). The findings revealed that the World Health Organization (WHO) safety checklist as recommended by the RCR was not utilized for any patient during the observed interventions. **Table-I** outlines the indicators recommended by the RCR for promoting safe sedation and anesthesia practice within radiology.

**Table-I:** Recommended indicators for the safe provision of sedation in radiology (Royal College of Radiologists).^4^

Indicator	Description
Training	The individual administering sedation must have appropriate and current training, consistent with local and national guidance.
Pre-procedure assessment	A documented pre-procedure assessment and sedation plan should be recorded in the patient’s notes.
Safety checklist	A completed WHO Surgical Safety Checklist, including sign-in and sign-out, must be available for every case.
Monitoring	Appropriate physiological monitoring should be applied in all cases, with observations recorded legibly and at appropriate intervals.
Resuscitation readiness	The resuscitation trolley and drug inventory must be checked daily and signed off.
Handover and discharge	A documented post-procedure handover and written discharge information should be available for every patient.

*Adapted from:* Royal College of Radiologists. Standards for the safe administration of sedation by radiologists. London: RCR; 2020.

### Development of a Modified WHO Safety Checklist for Radiology:

The outcomes of the audit were first presented at the Hospital Quality Council meeting and subsequently discussed in detail during a departmental meeting. Based on the findings, the lead author advocated for the adoption of an adapted or modified WHO safety checklist specifically designed for radiology procedures requiring sedation and anesthesia outside the operation theater. At the time, it was noted that both the endoscopy and bronchoscopy units within the institution were already utilizing specialty-specific checklists. This highlighted the feasibility and potential benefits of implementing a similar tool in Radiology to improve procedural standardization and strengthen patient safety.

Extensive discussions followed through email correspondence involving the Director of the Quality Department, the Medical Directors of both the Lahore and Peshawar centers, and the Head of the Anesthesia Department. A consensus was ultimately reached to move forward with the proposed initiative, and the development of a modified checklist was formally undertaken.

The design of the checklist prioritized conciseness while ensuring that all critical elements contributing to patient safety were incorporated. Redundant steps that overlapped with existing departmental practices were intentionally avoided to improve usability. Particular emphasis was placed on the identification of high-risk patients within the Radiology setting of a cancer hospital, as well as the integration of procedural steps that had previously been overlooked. These additions were informed by reports submitted to the Quality Department, which documented instances of near misses and critical incidents in the preceding months.

**Fig.1:**
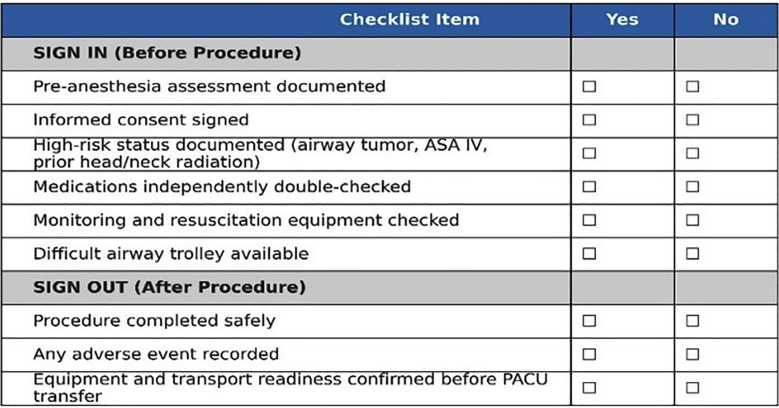
Modified WHO Safety Checklist for sedation and anesthesia outside the operating theatre. The checklist was adapted from the WHO Surgical Safety Checklist and modified on the basis of audit findings and recommendations by the Royal College of Anaesthetists (RCoA).

The final version of the modified WHO safety checklist was subsequently shared with the Trust leadership for review and endorsement, marking an important milestone in advancing patient safety practices during provision of anesthesia and sedation services within the Radiology department. The finalized checklist is presented in **Fig.1**.

## CONCLUSION

In summary, the promotion of a strong patient safety culture is increasingly vital given the rising complexity and volume of patients undergoing procedures beyond the traditional operating theater. The directives of the Francis Report emphasize the indispensable role of standards, regular audits, and structured training in ensuring the safe and effective delivery of healthcare. Preventing medical errors through the adoption of robust systems and strengthening organizational safety culture remains paramount.

Unfortunately, the current landscape of patient safety data in Pakistan is limited, particularly in relation to high-risk patient populations. The absence of a comprehensive national database on unexpected morbidity and mortality further underscores the urgent need for enhanced safety protocols and systematic reporting mechanisms.

At our institution, the extension of anesthesia services to radiology suites required a deliberate commitment to quality improvement. In line with professional body recommendations, annual audits were initiated to identify deficiencies and guide targeted interventions. This collaborative effort between institutional leadership and clinical departments resulted in the development of a modified WHO safety checklist tailored to the radiology settings.

The checklist was designed to be concise, evidence-based, and practical, ensuring the inclusion of critical safety steps while minimizing redundancy with existing workflows. Emphasis on high-risk patient identification and the incorporation of lessons learned from previous near misses reflected a proactive approach to addressing safety gaps.

Continued vigilance, regular audits, and adherence to standardized safety protocols will be essential to sustaining progress, reducing preventable harm, and upholding the highest standards of care in modern medical practice.
